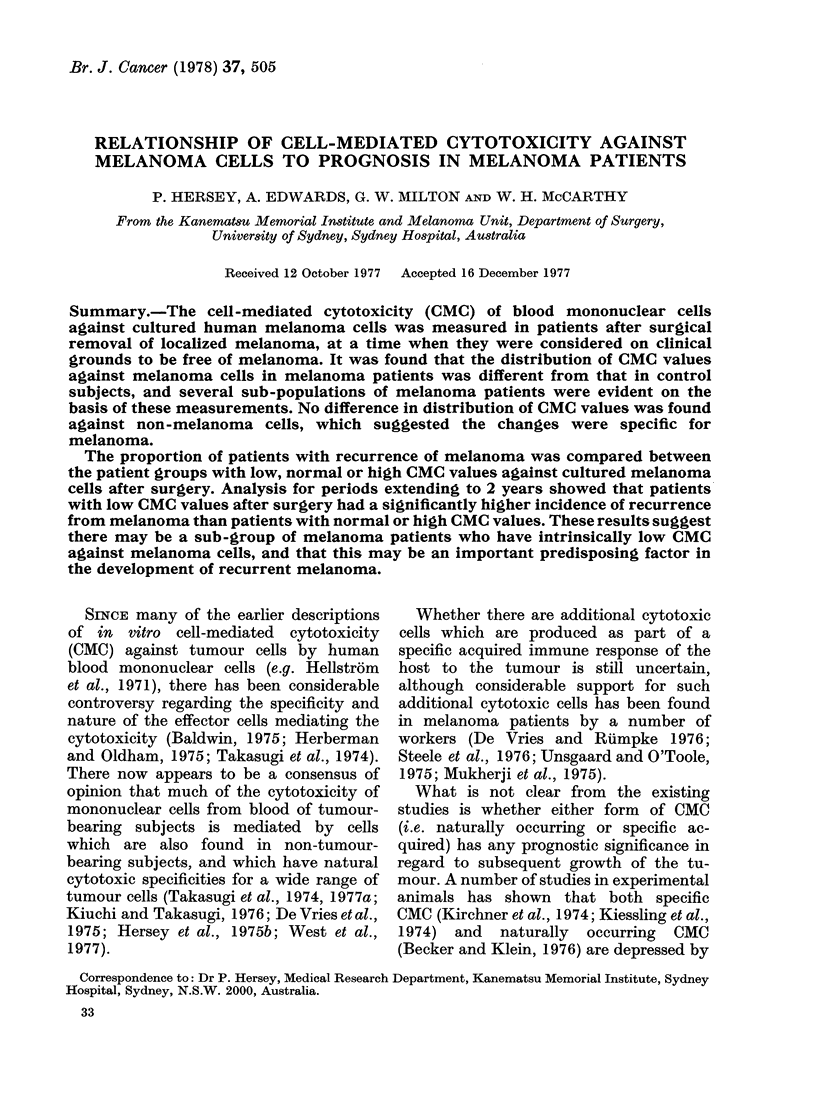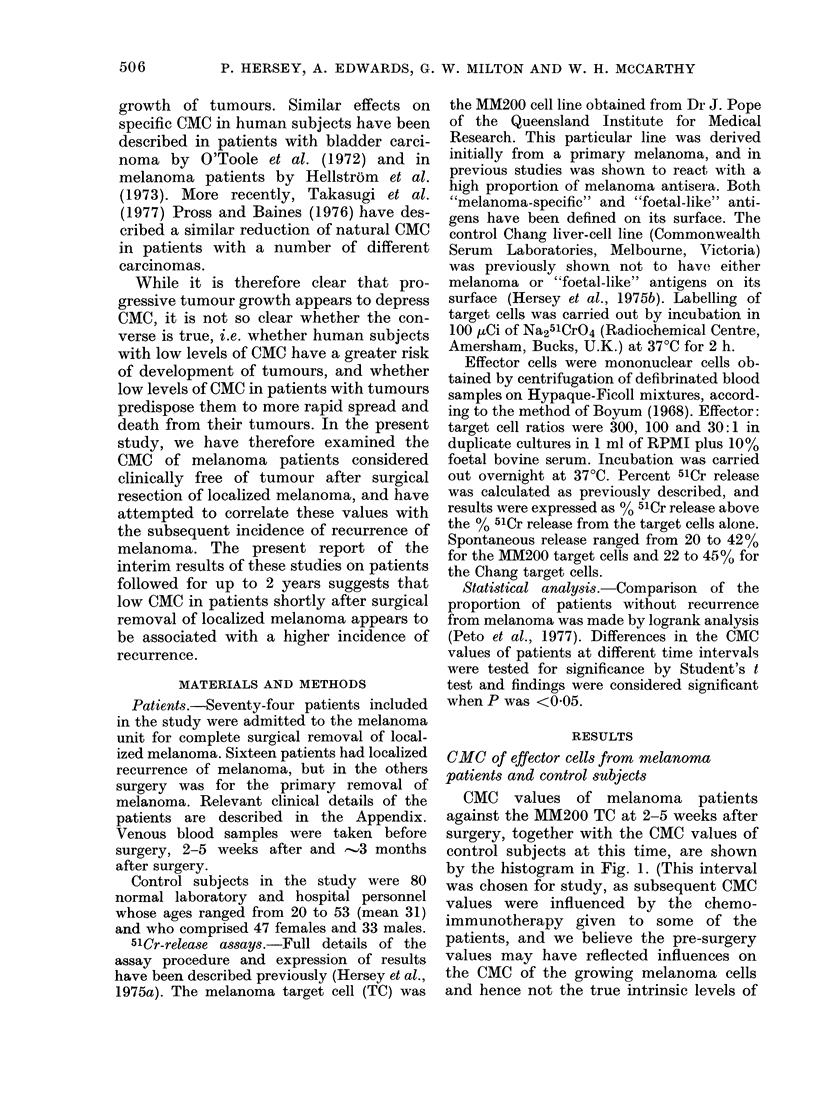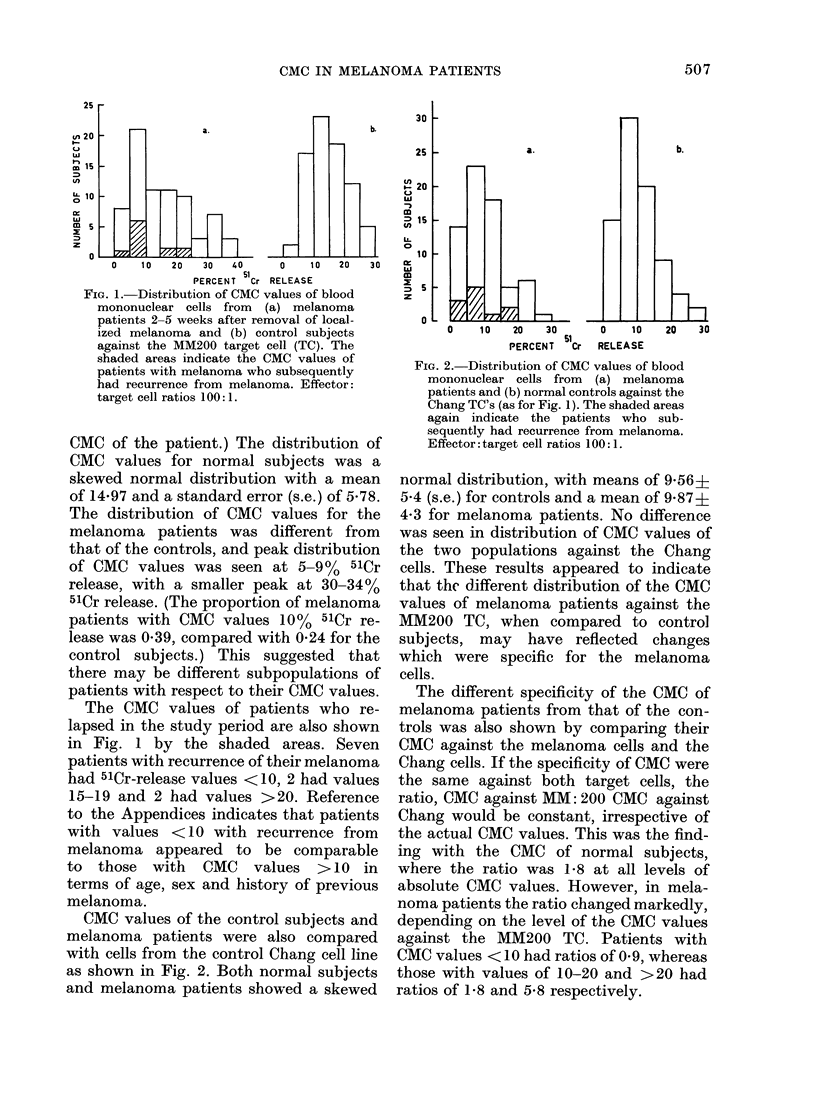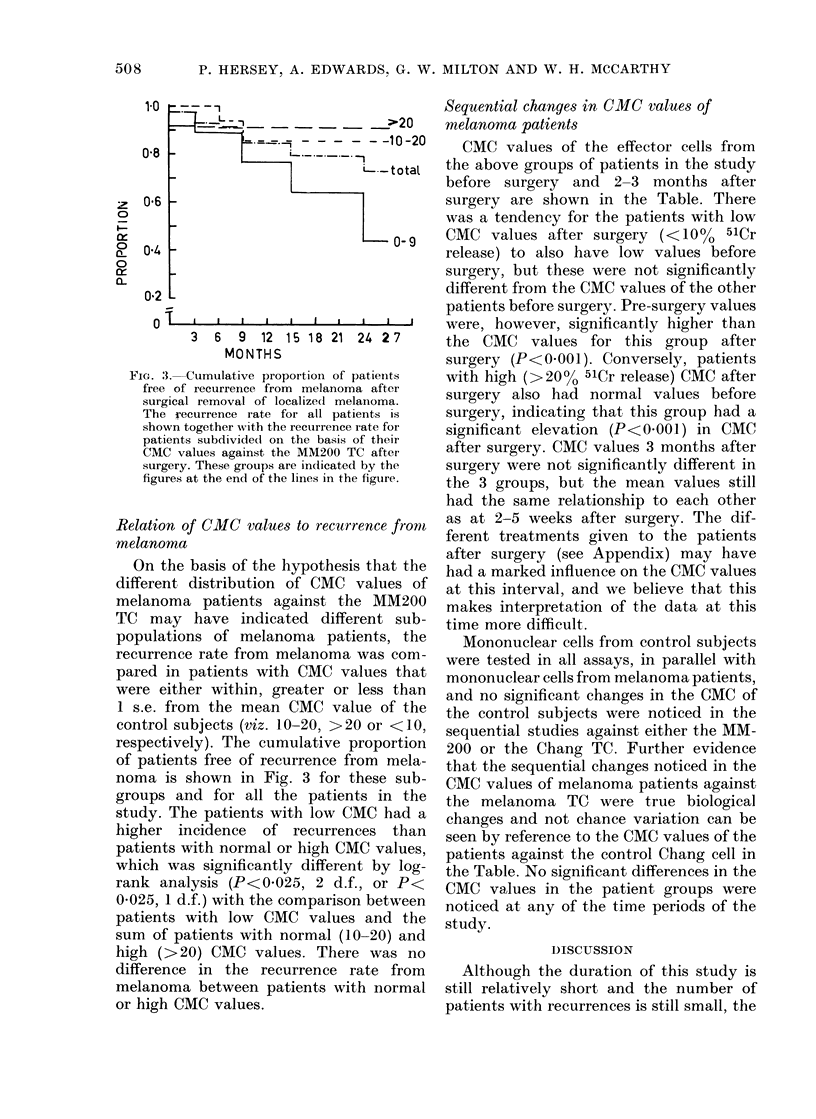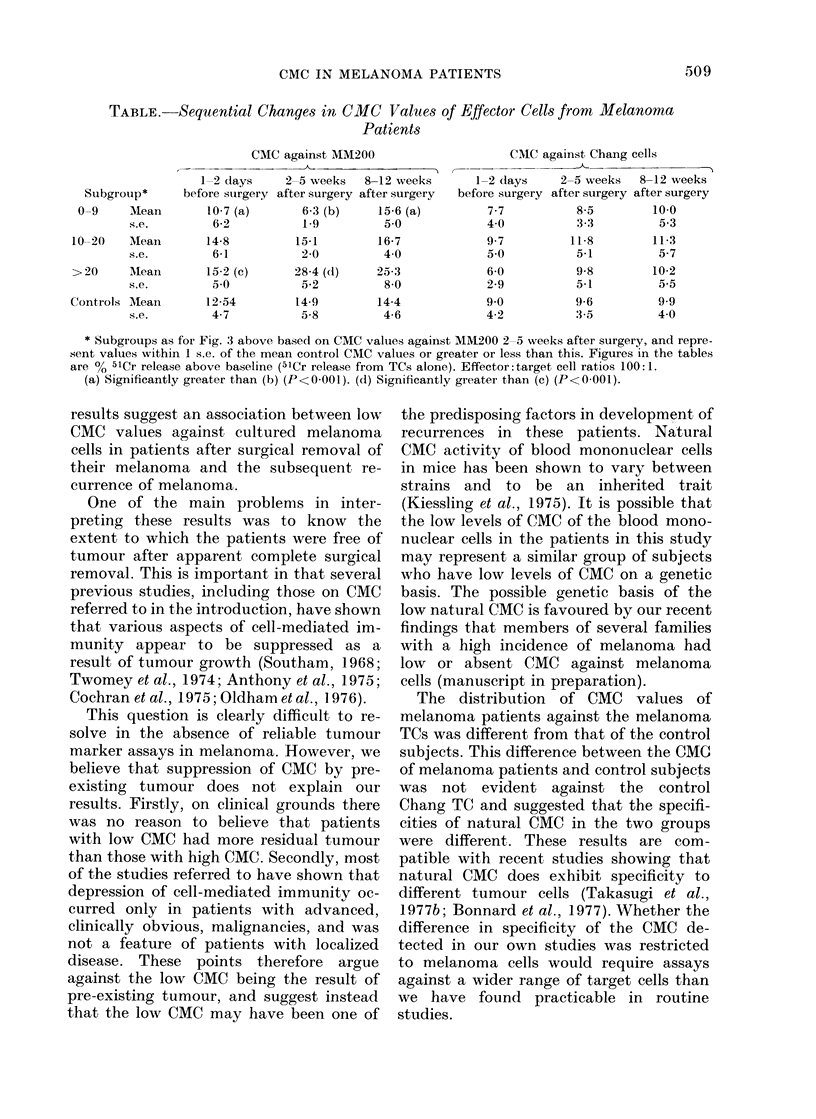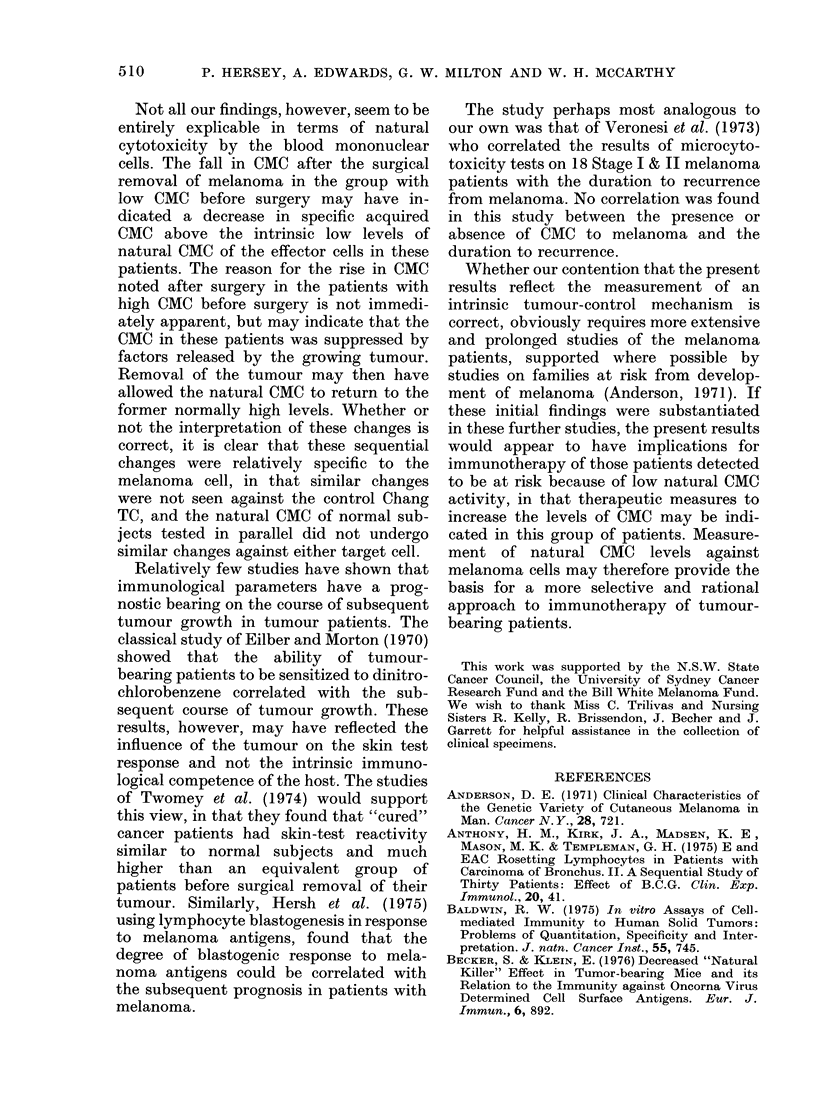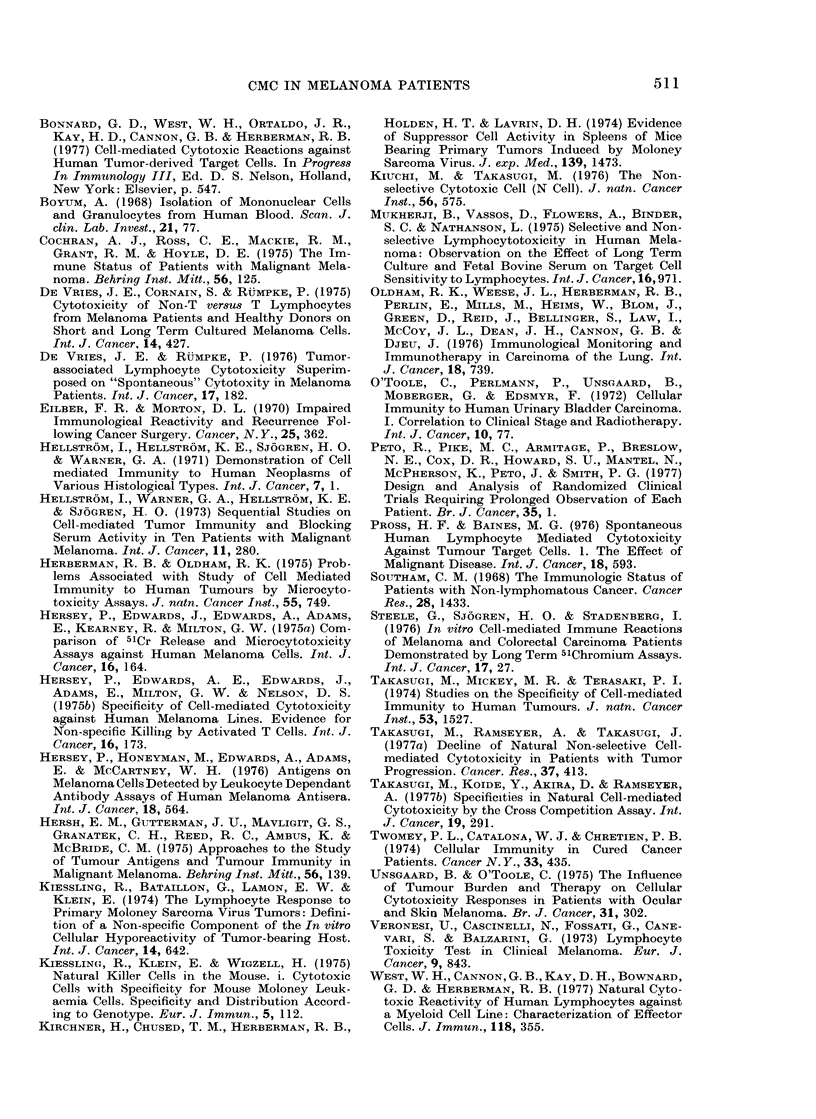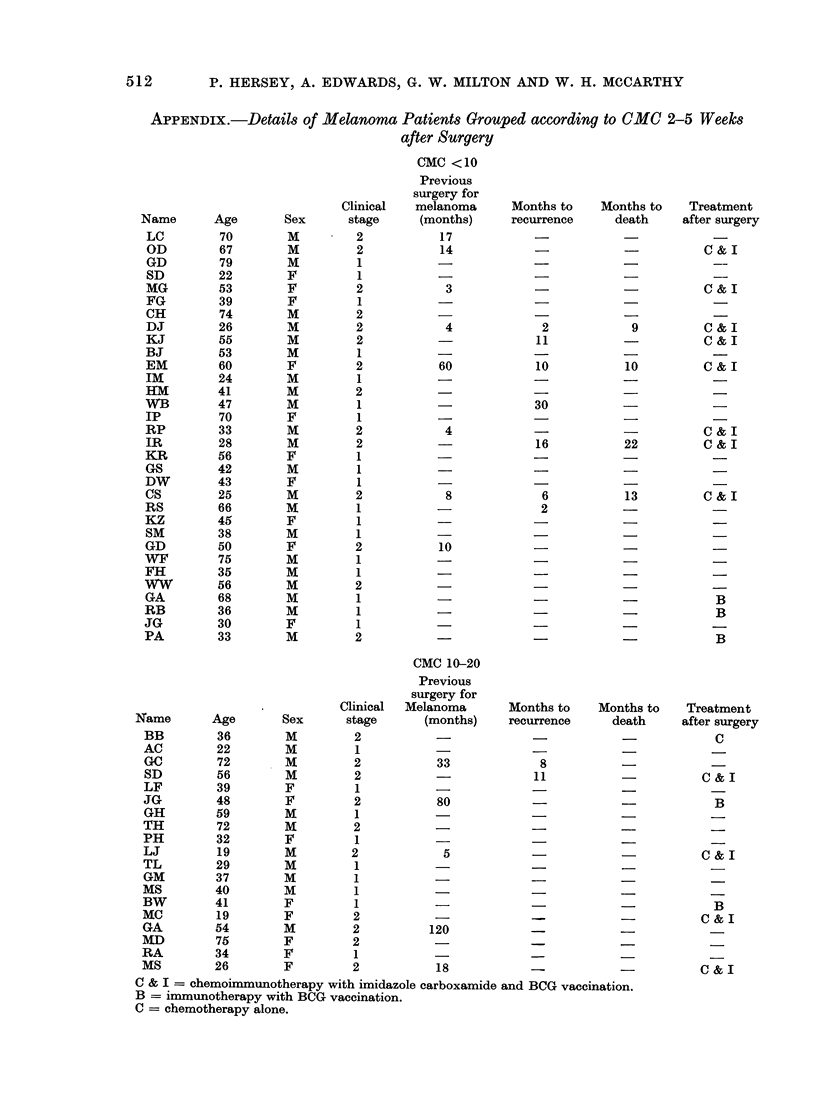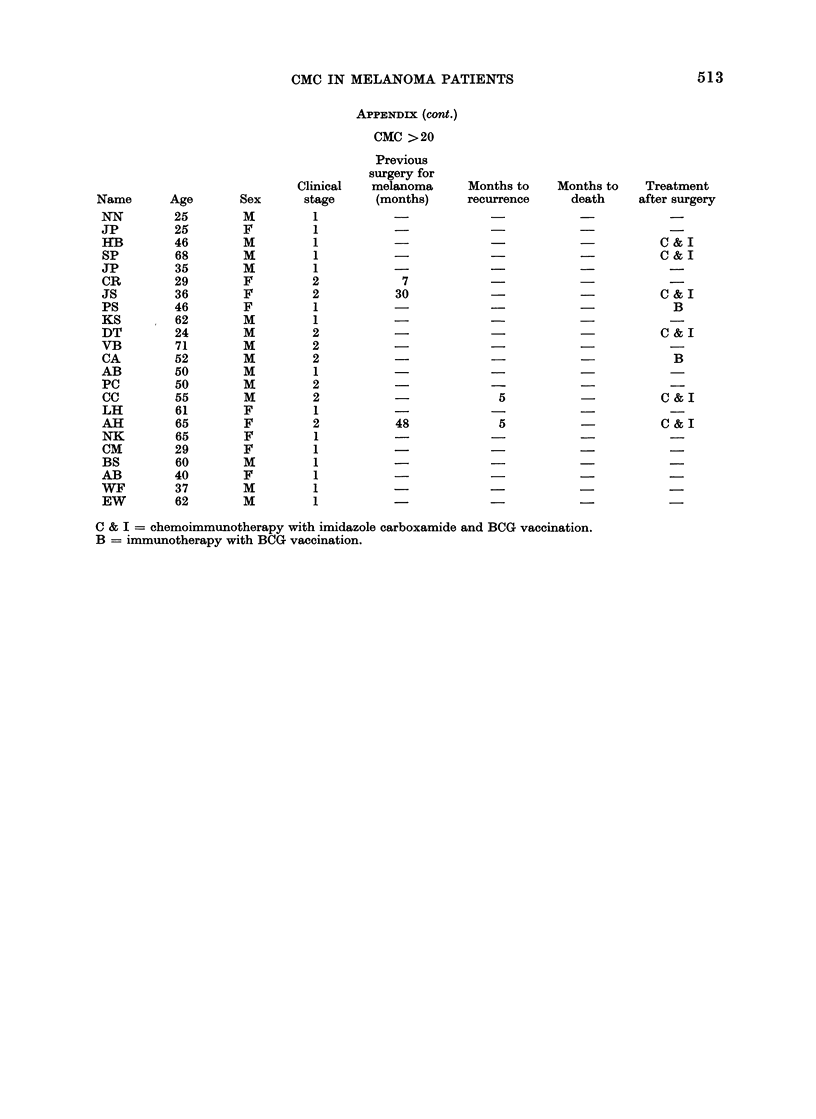# Relationship of cell-mediated cytotoxicity against melanoma cells to prognosis in melanoma patients.

**DOI:** 10.1038/bjc.1978.78

**Published:** 1978-04

**Authors:** P. Hershey, A. Edwards, G. W. Milton, W. H. McCarthy

## Abstract

The cell-mediated cytotoxicity (CMC) of blood mononuclear cells against cultured human melanoma cells was measured in patients after surgical removal of localized melanoma, at a time when they were considered on clinical grounds to be free of melanoma. It was found that the distribution of CMC values against melanoma cells in melanoma patients was different from that in control subjects, and several sub-populations of melanoma patients were evident on the basis of these measurements. No difference in distribution of CMC values was found against non-melanoma cells, which suggested the changes were specific for melanoma. The proportion of patients with recurrence of melanoma was compared between the patient groups with low, normal or high CMC values against cultured melanoma cells after surgery. Analysis for periods extending to 2 years showed that patients with low CMC values after surgery had a significantly higher incidence of recurrence from melanoma than patients with normal or high CMC values. These results suggest there may be a sub-group of melanoma patients who have intrinsically low CMC against melanoma cells, and that this may be an important predisposing factor in the development of recurrent melanoma.


*Br. J. Cancer* (1978) **37**, 505

### RELATIONSHIP OF CELL-MEDIATED CYTOTOXICITY AGAINST MELANOMA CELLS TO PROGNOSIS IN MELANOMA PATIENTS

P. HERSEY, A. EDWARDS, G. W. MILTON AND W. H. McCARTHY

*From the Kanematsu Memorial Institute and Melanoma Unit, Department of Surgery, University of Sydney, Sydney Hospital, Australia*

Received 12 October 1977 Accepted 16 December 1977

**Summary.—The cell-mediated cytotoxicity (CMC) of blood mononuclear cells against cultured human melanoma cells was measured in patients after surgical removal of localized melanoma, at a time when they were considered on clinical grounds to be free of melanoma. It was found that the distribution of CMC values against melanoma cells in melanoma patients was different from that in control subjects, and several sub-populations of melanoma patients were evident on the basis of these measurements. No difference in distribution of CMC values was found against non-melanoma cells, which suggested the changes were specific for melanoma.**

**The proportion of patients with recurrence of melanoma was compared between the patient groups with low, normal or high CMC values against cultured melanoma cells after surgery. Analysis for periods extending to 2 years showed that patients with low CMC values after surgery had a significantly higher incidence of recurrence from melanoma than patients with normal or high CMC values. These results suggest there may be a sub-group of melanoma patients who have intrinsically low CMC against melanoma cells, and that this may be an important predisposing factor in the development of recurrent melanoma.**

SINCE many of the earlier descriptions of *in vitro* cell-mediated cytotoxicity (CMC) against tumour cells by human blood mononuclear cells (*e.g.* Hellström *et al.*, 1971), there has been considerable controversy regarding the specificity and nature of the effector cells mediating the cytotoxicity (Baldwin, 1975; Herberman and Oldham, 1975; Takasugi *et al.*, 1974). There now appears to be a consensus of opinion that much of the cytotoxicity of mononuclear cells from blood of tumour-bearing subjects is mediated by cells which are also found in non-tumour-bearing subjects, and which have natural cytotoxic specificities for a wide range of tumour cells (Takasugi *et al.*, 1974, 1977*a*; Kiuchi and Takasugi, 1976; De Vries *et al.*, 1975; Hersey *et al.*, 1975*b*; West *et al.*, 1977).

Whether there are additional cytotoxic cells which are produced as part of a specific acquired immune response of the host to the tumour is still uncertain, although considerable support for such additional cytotoxic cells has been found in melanoma patients by a number of workers (De Vries and Rümpke 1976; Steele *et al.*, 1976; Unsgaard and O'Toole, 1975; Mukherji *et al.*, 1975).

What is not clear from the existing studies is whether either form of CMC (*i.e.* naturally occurring or specific acquired) has any prognostic significance in regard to subsequent growth of the tumour. A number of studies in experimental animals has shown that both specific CMC (Kirchner *et al.*, 1974; Kiessling *et al.*, 1974) and naturally occurring CMC (Becker and Klein, 1976) are depressed by

Correspondence to: Dr P. Hersey, Medical Research Department, Kanematsu Memorial Institute, Sydney Hospital, Sydney, N.S.W. 2000, Australia.

growth of tumours. Similar effects on specific CMC in human subjects have been described in patients with bladder carcinoma by O'Toole *et al.* (1972) and in melanoma patients by Hellström *et al.* (1973). More recently, Takasugi *et al.* (1977) Pross and Baines (1976) have described a similar reduction of natural CMC in patients with a number of different carcinomas.

While it is therefore clear that progressive tumour growth appears to depress CMC, it is not so clear whether the converse is true, *i.e.* whether human subjects with low levels of CMC have a greater risk of development of tumours, and whether low levels of CMC in patients with tumours predispose them to more rapid spread and death from their tumours. In the present study, we have therefore examined the CMC of melanoma patients considered clinically free of tumour after surgical resection of localized melanoma, and have attempted to correlate these values with the subsequent incidence of recurrence of melanoma. The present report of the interim results of these studies on patients followed for up to 2 years suggests that low CMC in patients shortly after surgical removal of localized melanoma appears to be associated with a higher incidence of recurrence.

### MATERIALS AND METHODS

*Patients.*—Seventy-four patients included in the study were admitted to the melanoma unit for complete surgical removal of localized melanoma. Sixteen patients had localized recurrence of melanoma, but in the others surgery was for the primary removal of melanoma. Relevant clinical details of the patients are described in the Appendix. Venous blood samples were taken before surgery, 2–5 weeks after and $\sim$3 months after surgery.

Control subjects in the study were 80 normal laboratory and hospital personnel whose ages ranged from 20 to 53 (mean 31) and who comprised 47 females and 33 males.

*$^{51}$Cr-release assays.*—Full details of the assay procedure and expression of results have been described previously (Hersey *et al.*, 1975*a*). The melanoma target cell (TC) was the MM200 cell line obtained from Dr J. Pope of the Queensland Institute for Medical Research. This particular line was derived initially from a primary melanoma, and in previous studies was shown to react with a high proportion of melanoma antisera. Both "melanoma-specific" and "foetal-like" antigens have been defined on its surface. The control Chang liver-cell line (Commonwealth Serum Laboratories, Melbourne, Victoria) was previously shown not to have either melanoma or "foetal-like" antigens on its surface (Hersey *et al.*, 1975*b*). Labelling of target cells was carried out by incubation in 100 $\mu$Ci of $Na_2{}^{51}CrO_4$ (Radiochemical Centre, Amersham, Bucks, U.K.) at 37°C for 2 h.

Effector cells were mononuclear cells obtained by centrifugation of defibrinated blood samples on Hypaque-Ficoll mixtures, according to the method of Boyum (1968). Effector: target cell ratios were 300, 100 and 30:1 in duplicate cultures in 1 ml of RPMI plus 10% foetal bovine serum. Incubation was carried out overnight at 37°C. Percent $^{51}$Cr release was calculated as previously described, and results were expressed as % $^{51}$Cr release above the % $^{51}$Cr release from the target cells alone. Spontaneous release ranged from 20 to 42% for the MM200 target cells and 22 to 45% for the Chang target cells.

*Statistical analysis.*—Comparison of the proportion of patients without recurrence from melanoma was made by logrank analysis (Peto *et al.*, 1977). Differences in the CMC values of patients at different time intervals were tested for significance by Student's *t* test and findings were considered significant when $P$ was $<0{\cdot}05$.

### RESULTS

### *CMC of effector cells from melanoma patients and control subjects*

CMC values of melanoma patients against the MM200 TC at 2–5 weeks after surgery, together with the CMC values of control subjects at this time, are shown by the histogram in Fig. 1. (This interval was chosen for study, as subsequent CMC values were influenced by the chemo-immunotherapy given to some of the patients, and we believe the pre-surgery values may have reflected influences on the CMC of the growing melanoma cells and hence not the true intrinsic levels of

FIG. 1.—Distribution of CMC values of blood mononuclear cells from (a) melanoma patients 2–5 weeks after removal of localized melanoma and (b) control subjects against the MM200 target cell (TC). The shaded areas indicate the CMC values of patients with melanoma who subsequently had recurrence from melanoma. Effector: target cell ratios 100:1.

FIG. 2.—Distribution of CMC values of blood mononuclear cells from (a) melanoma patients and (b) normal controls against the Chang TC's (as for Fig. 1). The shaded areas again indicate the patients who subsequently had recurrence from melanoma. Effector:target cell ratios 100:1.

CMC of the patient.) The distribution of CMC values for normal subjects was a skewed normal distribution with a mean of 14·97 and a standard error (s.e.) of 5·78. The distribution of CMC values for the melanoma patients was different from that of the controls, and peak distribution of CMC values was seen at 5–9% $^{51}Cr$ release, with a smaller peak at 30–34% $^{51}Cr$ release. (The proportion of melanoma patients with CMC values 10% $^{51}Cr$ release was 0·39, compared with 0·24 for the control subjects.) This suggested that there may be different subpopulations of patients with respect to their CMC values.

The CMC values of patients who relapsed in the study period are also shown in Fig. 1 by the shaded areas. Seven patients with recurrence of their melanoma had $^{51}Cr$-release values $<10$, 2 had values 15–19 and 2 had values $>20$. Reference to the Appendices indicates that patients with values $<10$ with recurrence from melanoma appeared to be comparable to those with CMC values $>10$ in terms of age, sex and history of previous melanoma.

CMC values of the control subjects and melanoma patients were also compared with cells from the control Chang cell line as shown in Fig. 2. Both normal subjects and melanoma patients showed a skewed normal distribution, with means of 9·56 ± 5·4 (s.e.) for controls and a mean of 9·87 ± 4·3 for melanoma patients. No difference was seen in distribution of CMC values of the two populations against the Chang cells. These results appeared to indicate that thc different distribution of the CMC values of melanoma patients against the MM200 TC, when compared to control subjects, may have reflected changes which were specific for the melanoma cells.

The different specificity of the CMC of melanoma patients from that of the controls was also shown by comparing their CMC against the melanoma cells and the Chang cells. If the specificity of CMC were the same against both target cells, the ratio, CMC against MM: 200 CMC against Chang would be constant, irrespective of the actual CMC values. This was the finding with the CMC of normal subjects, where the ratio was 1·8 at all levels of absolute CMC values. However, in melanoma patients the ratio changed markedly, depending on the level of the CMC values against the MM200 TC. Patients with CMC values $<10$ had ratios of 0·9, whereas those with values of 10–20 and $>20$ had ratios of 1·8 and 5·8 respectively.

FIG. 3.—Cumulative proportion of patients free of recurrence from melanoma after surgical removal of localized melanoma. The recurrence rate for all patients is shown together with the recurrence rate for patients subdivided on the basis of their CMC values against the MM200 TC after surgery. These groups are indicated by the figures at the end of the lines in the figure.

*Relation of CMC values to recurrence from melanoma*

On the basis of the hypothesis that the different distribution of CMC values of melanoma patients against the MM200 TC may have indicated different subpopulations of melanoma patients, the recurrence rate from melanoma was compared in patients with CMC values that were either within, greater or less than 1 s.e. from the mean CMC value of the control subjects (*viz.* 10–20, >20 or <10, respectively). The cumulative proportion of patients free of recurrence from melanoma is shown in Fig. 3 for these subgroups and for all the patients in the study. The patients with low CMC had a higher incidence of recurrences than patients with normal or high CMC values, which was significantly different by log-rank analysis ($P<0{\cdot}025$, 2 d.f., or $P<0{\cdot}025$, 1 d.f.) with the comparison between patients with low CMC values and the sum of patients with normal (10–20) and high (>20) CMC values. There was no difference in the recurrence rate from melanoma between patients with normal or high CMC values.

*Sequential changes in CMC values of melanoma patients*

CMC values of the effector cells from the above groups of patients in the study before surgery and 2–3 months after surgery are shown in the Table. There was a tendency for the patients with low CMC values after surgery (<10% $^{51}Cr$ release) to also have low values before surgery, but these were not significantly different from the CMC values of the other patients before surgery. Pre-surgery values were, however, significantly higher than the CMC values for this group after surgery ($P<0{\cdot}001$). Conversely, patients with high (>20% $^{51}Cr$ release) CMC after surgery also had normal values before surgery, indicating that this group had a significant elevation ($P<0{\cdot}001$) in CMC after surgery. CMC values 3 months after surgery were not significantly different in the 3 groups, but the mean values still had the same relationship to each other as at 2–5 weeks after surgery. The different treatments given to the patients after surgery (see Appendix) may have had a marked influence on the CMC values at this interval, and we believe that this makes interpretation of the data at this time more difficult.

Mononuclear cells from control subjects were tested in all assays, in parallel with mononuclear cells from melanoma patients, and no significant changes in the CMC of the control subjects were noticed in the sequential studies against either the MM-200 or the Chang TC. Further evidence that the sequential changes noticed in the CMC values of melanoma patients against the melanoma TC were true biological changes and not chance variation can be seen by reference to the CMC values of the patients against the control Chang cell in the Table. No significant differences in the CMC values in the patient groups were noticed at any of the time periods of the study.

### DISCUSSION

Although the duration of this study is still relatively short and the number of patients with recurrences is still small, the

TABLE.—*Sequential Changes in CMC Values of Effector Cells from Melanoma Patients*

| Subgroup* | | CMC against MM200 1–2 days before surgery | CMC against MM200 2–5 weeks after surgery | CMC against MM200 8–12 weeks after surgery | CMC against Chang cells 1–2 days before surgery | CMC against Chang cells 2–5 weeks after surgery | CMC against Chang cells 8–12 weeks after surgery |
|---|---|---|---|---|---|---|---|
| 0–9 | Mean | 10·7 (a) | 6·3 (b) | 15·6 (a) | 7·7 | 8·5 | 10·0 |
| | s.e. | 6·2 | 1·9 | 5·0 | 4·0 | 3·3 | 5·3 |
| 10–20 | Mean | 14·8 | 15·1 | 16·7 | 9·7 | 11·8 | 11·3 |
| | s.e. | 6·1 | 2·0 | 4·0 | 5·0 | 5·1 | 5·7 |
| >20 | Mean | 15·2 (c) | 28·4 (d) | 25·3 | 6·0 | 9·8 | 10·2 |
| | s.e. | 5·0 | 5·2 | 8·0 | 2·9 | 5·1 | 5·5 |
| Controls | Mean | 12·54 | 14·9 | 14·4 | 9·0 | 9·6 | 9·9 |
| | s.e. | 4·7 | 5·8 | 4·6 | 4·2 | 3·5 | 4·0 |

* Subgroups as for Fig. 3 above based on CMC values against MM200 2–5 weeks after surgery, and represent values within 1 s.e. of the mean control CMC values or greater or less than this. Figures in the tables are % $^{51}$Cr release above baseline ($^{51}$Cr release from TCs alone). Effector:target cell ratios 100:1.
(a) Significantly greater than (b) ($P<0·001$). (d) Significantly greater than (c) ($P<0·001$).

results suggest an association between low CMC values against cultured melanoma cells in patients after surgical removal of their melanoma and the subsequent recurrence of melanoma.

One of the main problems in interpreting these results was to know the extent to which the patients were free of tumour after apparent complete surgical removal. This is important in that several previous studies, including those on CMC referred to in the introduction, have shown that various aspects of cell-mediated immunity appear to be suppressed as a result of tumour growth (Southam, 1968; Twomey *et al.*, 1974; Anthony *et al.*, 1975; Cochran *et al.*, 1975; Oldham *et al.*, 1976).

This question is clearly difficult to resolve in the absence of reliable tumour marker assays in melanoma. However, we believe that suppression of CMC by pre-existing tumour does not explain our results. Firstly, on clinical grounds there was no reason to believe that patients with low CMC had more residual tumour than those with high CMC. Secondly, most of the studies referred to have shown that depression of cell-mediated immunity occurred only in patients with advanced, clinically obvious, malignancies, and was not a feature of patients with localized disease. These points therefore argue against the low CMC being the result of pre-existing tumour, and suggest instead that the low CMC may have been one of the predisposing factors in development of recurrences in these patients. Natural CMC activity of blood mononuclear cells in mice has been shown to vary between strains and to be an inherited trait (Kiessling *et al.*, 1975). It is possible that the low levels of CMC of the blood mononuclear cells in the patients in this study may represent a similar group of subjects who have low levels of CMC on a genetic basis. The possible genetic basis of the low natural CMC is favoured by our recent findings that members of several families with a high incidence of melanoma had low or absent CMC against melanoma cells (manuscript in preparation).

The distribution of CMC values of melanoma patients against the melanoma TCs was different from that of the control subjects. This difference between the CMC of melanoma patients and control subjects was not evident against the control Chang TC and suggested that the specificities of natural CMC in the two groups were different. These results are compatible with recent studies showing that natural CMC does exhibit specificity to different tumour cells (Takasugi *et al.*, 1977*b*; Bonnard *et al.*, 1977). Whether the difference in specificity of the CMC detected in our own studies was restricted to melanoma cells would require assays against a wider range of target cells than we have found practicable in routine studies.

Not all our findings, however, seem to be entirely explicable in terms of natural cytotoxicity by the blood mononuclear cells. The fall in CMC after the surgical removal of melanoma in the group with low CMC before surgery may have indicated a decrease in specific acquired CMC above the intrinsic low levels of natural CMC of the effector cells in these patients. The reason for the rise in CMC noted after surgery in the patients with high CMC before surgery is not immediately apparent, but may indicate that the CMC in these patients was suppressed by factors released by the growing tumour. Removal of the tumour may then have allowed the natural CMC to return to the former normally high levels. Whether or not the interpretation of these changes is correct, it is clear that these sequential changes were relatively specific to the melanoma cell, in that similar changes were not seen against the control Chang TC, and the natural CMC of normal subjects tested in parallel did not undergo similar changes against either target cell.

Relatively few studies have shown that immunological parameters have a prognostic bearing on the course of subsequent tumour growth in tumour patients. The classical study of Eilber and Morton (1970) showed that the ability of tumour-bearing patients to be sensitized to dinitrochlorobenzene correlated with the subsequent course of tumour growth. These results, however, may have reflected the influence of the tumour on the skin test response and not the intrinsic immunological competence of the host. The studies of Twomey *et al.* (1974) would support this view, in that they found that "cured" cancer patients had skin-test reactivity similar to normal subjects and much higher than an equivalent group of patients before surgical removal of their tumour. Similarly, Hersh *et al.* (1975) using lymphocyte blastogenesis in response to melanoma antigens, found that the degree of blastogenic response to melanoma antigens could be correlated with the subsequent prognosis in patients with melanoma.

The study perhaps most analogous to our own was that of Veronesi *et al.* (1973) who correlated the results of microcytotoxicity tests on 18 Stage I & II melanoma patients with the duration to recurrence from melanoma. No correlation was found in this study between the presence or absence of CMC to melanoma and the duration to recurrence.

Whether our contention that the present results reflect the measurement of an intrinsic tumour-control mechanism is correct, obviously requires more extensive and prolonged studies of the melanoma patients, supported where possible by studies on families at risk from development of melanoma (Anderson, 1971). If these initial findings were substantiated in these further studies, the present results would appear to have implications for immunotherapy of those patients detected to be at risk because of low natural CMC activity, in that therapeutic measures to increase the levels of CMC may be indicated in this group of patients. Measurement of natural CMC levels against melanoma cells may therefore provide the basis for a more selective and rational approach to immunotherapy of tumour-bearing patients.

This work was supported by the N.S.W. State Cancer Council, the University of Sydney Cancer Research Fund and the Bill White Melanoma Fund. We wish to thank Miss C. Trilivas and Nursing Sisters R. Kelly, R. Brissendon, J. Becher and J. Garrett for helpful assistance in the collection of clinical specimens.

### REFERENCES

ANDERSON, D. E. (1971) Clinical Characteristics of the Genetic Variety of Cutaneous Melanoma in Man. *Cancer N.Y.*, **28**, 721.

ANTHONY, H. M., KIRK, J. A., MADSEN, K. E., MASON, M. K. & TEMPLEMAN, G. H. (1975) E and EAC Rosetting Lymphocytes in Patients with Carcinoma of Bronchus. II. A Sequential Study of Thirty Patients: Effect of B.C.G. *Clin. Exp. Immunol.*, **20**, 41.

BALDWIN, R. W. (1975) *In vitro* Assays of Cell-mediated Immunity to Human Solid Tumors: Problems of Quantitation, Specificity and Interpretation. *J. natn. Cancer Inst.*, **55**, 745.

BECKER, S. & KLEIN, E. (1976) Decreased "Natural Killer" Effect in Tumor-bearing Mice and its Relation to the Immunity against Oncorna Virus Determined Cell Surface Antigens. *Eur. J. Immun.*, **6**, 892.

BONNARD, G. D., WEST, W. H., ORTALDO, J. R., KAY, H. D., CANNON, G. B. & HERBERMAN, R. B. (1977) Cell-mediated Cytotoxic Reactions against Human Tumor-derived Target Cells. In *Progress In Immunology III*, Ed. D. S. Nelson, Holland, New York: Elsevier, p. 547.

BOYUM, A. (1968) Isolation of Mononuclear Cells and Granulocytes from Human Blood. *Scan. J. clin. Lab. Invest.*, **21**, 77.

COCHRAN, A. J., ROSS, C. E., MACKIE, R. M., GRANT, R. M. & HOYLE, D. E. (1975) The Immune Status of Patients with Malignant Melanoma. *Behring Inst. Mitt.*, **56**, 125.

DE VRIES, J. E., CORNAIN, S. & RÜMPKE, P. (1975) Cytotoxicity of Non-T *versus* T Lymphocytes from Melanoma Patients and Healthy Donors on Short and Long Term Cultured Melanoma Cells. *Int. J. Cancer*, **14**, 427.

DE VRIES, J. E. & RÜMPKE, P. (1976) Tumor-associated Lymphocyte Cytotoxicity Superimposed on "Spontaneous" Cytotoxity in Melanoma Patients. *Int. J. Cancer*, **17**, 182.

EILBER, F. R. & MORTON, D. L. (1970) Impaired Immunological Reactivity and Recurrence Following Cancer Surgery. *Cancer, N.Y.*, **25**, 362.

HELLSTRÖM, I., HELLSTRÖM, K. E., SJÖGREN, H. O. & WARNER, G. A. (1971) Demonstration of Cell mediated Immunity to Human Neoplasms of Various Histological Types. *Int. J. Cancer*, **7**, 1.

HELLSTRÖM, I., WARNER, G. A., HELLSTRÖM, K. E. & SJÖGREN, H. O. (1973) Sequential Studies on Cell-mediated Tumor Immunity and Blocking Serum Activity in Ten Patients with Malignant Melanoma. *Int. J. Cancer*, **11**, 280.

HERBERMAN, R. B. & OLDHAM, R. K. (1975) Problems Associated with Study of Cell Mediated Immunity to Human Tumours by Microcytotoxicity Assays. *J. natn. Cancer Inst.*, **55**, 749.

HERSEY, P., EDWARDS, J., EDWARDS, A., ADAMS, E., KEARNEY, R. & MILTON, G. W. (1975*a*) Comparison of $^{51}$Cr Release and Microcytotoxicity Assays against Human Melanoma Cells. *Int. J. Cancer*, **16**, 164.

HERSEY, P., EDWARDS, A. E., EDWARDS, J., ADAMS, E., MILTON, G. W. & NELSON, D. S. (1975*b*) Specificity of Cell-mediated Cytotoxicity against Human Melanoma Lines. Evidence for Non-specific Killing by Activated T Cells. *Int. J. Cancer*, **16**, 173.

HERSEY, P., HONEYMAN, M., EDWARDS, A., ADAMS, E. & MCCARTNEY, W. H. (1976) Antigens on Melanoma Cells Detected by Leukocyte Dependant Antibody Assays of Human Melanoma Antisera. *Int. J. Cancer*, **18**, 564.

HERSH, E. M., GUTTERMAN, J. U., MAVLIGIT, G. S., GRANATEK, C. H., REED, R. C., AMBUS, K. & MCBRIDE, C. M. (1975) Approaches to the Study of Tumour Antigens and Tumour Immunity in Malignant Melanoma. *Behring Inst. Mitt.*, **56**, 139.

KIESSLING, R., BATAILLON, G., LAMON, E. W. & KLEIN, E. (1974) The Lymphocyte Response to Primary Moloney Sarcoma Virus Tumors: Definition of a Non-specific Component of the *In vitro* Cellular Hyporeactivity of Tumor-bearing Host. *Int. J. Cancer*, **14**, 642.

KIESSLING, R., KLEIN, E. & WIGZELL, H. (1975) Natural Killer Cells in the Mouse. i. Cytotoxic Cells with Specificity for Mouse Moloney Leukaemia Cells. Specificity and Distribution According to Genotype. *Eur. J. Immun.*, **5**, 112.

KIRCHNER, H., CHUSED, T. M., HERBERMAN, R. B., HOLDEN, H. T. & LAVRIN, D. H. (1974) Evidence of Suppressor Cell Activity in Spleens of Mice Bearing Primary Tumors Induced by Moloney Sarcoma Virus. *J. exp. Med.*, **139**, 1473.

KIUCHI, M. & TAKASUGI, M. (1976) The Non-selective Cytotoxic Cell (N Cell). *J. natn. Cancer Inst.*, **56**, 575.

MUKHERJI, B., VASSOS, D., FLOWERS, A., BINDER, S. C. & NATHANSON, L. (1975) Selective and Non-selective Lymphocytotoxicity in Human Melanoma: Observation on the Effect of Long Term Culture and Fetal Bovine Serum on Target Cell Sensitivity to Lymphocytes. *Int. J. Cancer*, **16**, 971.

OLDHAM, R. K., WEESE, J. L., HERBERMAN, R. B., PERLIN, E., MILLS, M., HEIMS, W., BLOM, J., GREEN, D., REID, J., BELLINGER, S., LAW, I., MCCOY, J. L., DEAN, J. H., CANNON, G. B. & DJEU, J. (1976) Immunological Monitoring and Immunotherapy in Carcinoma of the Lung. *Int. J. Cancer*, **18**, 739.

O'TOOLE, C., PERLMANN, P., UNSGAARD, B., MOBERGER, G. & EDSMYR, F. (1972) Cellular Immunity to Human Urinary Bladder Carcinoma. I. Correlation to Clinical Stage and Radiotherapy. *Int. J. Cancer*, **10**, 77.

PETO, R., PIKE, M. C., ARMITAGE, P., BRESLOW, N. E., COX, D. R., HOWARD, S. U., MANTEL, N., MCPHERSON, K., PETO, J. & SMITH, P. G. (1977) Design and Analysis of Randomized Clinical Trials Requiring Prolonged Observation of Each Patient. *Br. J. Cancer*, **35**, 1.

PROSS, H. F. & BAINES, M. G. (976) Spontaneous Human Lymphocyte Mediated Cytotoxicity Against Tumour Target Cells. 1. The Effect of Malignant Disease. *Int. J. Cancer*, **18**, 593.

SOUTHAM, C. M. (1968) The Immunologic Status of Patients with Non-lymphomatous Cancer. *Cancer Res.*, **28**, 1433.

STEELE, G., SJÖGREN, H. O. & STADENBERG, I. (1976) *In vitro* Cell-mediated Immune Reactions of Melanoma and Colorectal Carcinoma Patients Demonstrated by Long Term $^{51}$Chromium Assays. *Int. J. Cancer*, **17**, 27.

TAKASUGI, M., MICKEY, M. R. & TERASAKI, P. I. (1974) Studies on the Specificity of Cell-mediated Immunity to Human Tumours. *J. natn. Cancer Inst.*, **53**, 1527.

TAKASUGI, M., RAMSEYER, A. & TAKASUGI, J. (1977*a*) Decline of Natural Non-selective Cell-mediated Cytotoxicity in Patients with Tumor Progression. *Cancer. Res.*, **37**, 413.

TAKASUGI, M., KOIDE, Y., AKIRA, D. & RAMSEYER, A. (1977*b*) Specificities in Natural Cell-mediated Cytotoxicity by the Cross Competition Assay. *Int. J. Cancer*, **19**, 291.

TWOMEY, P. L., CATALONA, W. J. & CHRETIEN, P. B. (1974) Cellular Immunity in Cured Cancer Patients. *Cancer N.Y.*, **33**, 435.

UNSGAARD, B. & O'TOOLE, C. (1975) The Influence of Tumour Burden and Therapy on Cellular Cytotoxicity Responses in Patients with Ocular and Skin Melanoma. *Br. J. Cancer*, **31**, 302.

VERONESI, U., CASCINELLI, N., FOSSATI, G., CANEVARI, S. & BALZARINI, G. (1973) Lymphocyte Toxicity Test in Clinical Melanoma. *Eur. J. Cancer*, **9**, 843.

WEST, W. H., CANNON, G. B., KAY, D. H., BOWNARD, G. D. & HERBERMAN, R. B. (1977) Natural Cytotoxic Reactivity of Human Lymphocytes against a Myeloid Cell Line: Characterization of Effector Cells. *J. Immun.*, **118**, 355.

APPENDIX.—*Details of Melanoma Patients Grouped according to CMC 2–5 Weeks after Surgery*

CMC <10

| Name | Age | Sex | Clinical stage | Previous surgery for melanoma (months) | Months to recurrence | Months to death | Treatment after surgery |
|---|---|---|---|---|---|---|---|
| LC | 70 | M | 2 | 17 | — | — | — |
| OD | 67 | M | 2 | 14 | — | — | C & I |
| GD | 79 | M | 1 | — | — | — | — |
| SD | 22 | F | 1 | — | — | — | — |
| MG | 53 | F | 2 | 3 | — | — | C & I |
| FG | 39 | F | 1 | — | — | — | — |
| CH | 74 | M | 2 | — | — | — | — |
| DJ | 26 | M | 2 | 4 | 2 | 9 | C & I |
| KJ | 55 | M | 2 | — | 11 | — | C & I |
| BJ | 53 | M | 1 | — | — | — | — |
| EM | 60 | F | 2 | 60 | 10 | 10 | C & I |
| IM | 24 | M | 1 | — | — | — | — |
| HM | 41 | M | 2 | — | — | — | — |
| WB | 47 | M | 1 | — | 30 | — | — |
| IP | 70 | F | 1 | — | — | — | — |
| RP | 33 | M | 2 | 4 | — | — | C & I |
| IR | 28 | M | 2 | — | 16 | 22 | C & I |
| KR | 56 | F | 1 | — | — | — | — |
| GS | 42 | M | 1 | — | — | — | — |
| DW | 43 | F | 1 | — | — | — | — |
| CS | 25 | M | 2 | 8 | 6 | 13 | C & I |
| RS | 66 | M | 1 | — | 2 | — | — |
| KZ | 45 | F | 1 | — | — | — | — |
| SM | 38 | M | 1 | — | — | — | — |
| GD | 50 | F | 2 | 10 | — | — | — |
| WF | 75 | M | 1 | — | — | — | — |
| FH | 35 | M | 1 | — | — | — | — |
| WW | 56 | M | 2 | — | — | — | — |
| GA | 68 | M | 1 | — | — | — | B |
| RB | 36 | M | 1 | — | — | — | B |
| JG | 30 | F | 1 | — | — | — | — |
| PA | 33 | M | 2 | — | — | — | B |

CMC 10–20

| Name | Age | Sex | Clinical stage | Previous surgery for Melanoma (months) | Months to recurrence | Months to death | Treatment after surgery |
|---|---|---|---|---|---|---|---|
| BB | 36 | M | 2 | — | — | — | C |
| AC | 22 | M | 1 | — | — | — | — |
| GC | 72 | M | 2 | 33 | 8 | — | — |
| SD | 56 | M | 2 | — | 11 | — | C & I |
| LF | 39 | F | 1 | — | — | — | — |
| JG | 48 | F | 2 | 80 | — | — | B |
| GH | 59 | M | 1 | — | — | — | — |
| TH | 72 | M | 2 | — | — | — | — |
| PH | 32 | F | 1 | — | — | — | — |
| LJ | 19 | M | 2 | 5 | — | — | C & I |
| TL | 29 | M | 1 | — | — | — | — |
| GM | 37 | M | 1 | — | — | — | — |
| MS | 40 | M | 1 | — | — | — | — |
| BW | 41 | F | 1 | — | — | — | B |
| MC | 19 | F | 2 | — | — | — | C & I |
| GA | 54 | M | 2 | 120 | — | — | — |
| MD | 75 | F | 2 | — | — | — | — |
| RA | 34 | F | 1 | — | — | — | — |
| MS | 26 | F | 2 | 18 | — | — | C & I |

C & I = chemoimmunotherapy with imidazole carboxamide and BCG vaccination.
B = immunotherapy with BCG vaccination.
C = chemotherapy alone.

APPENDIX (*cont.*)

CMC >20

| Name | Age | Sex | Clinical stage | Previous surgery for melanoma (months) | Months to recurrence | Months to death | Treatment after surgery |
|---|---|---|---|---|---|---|---|
| NN | 25 | M | 1 | — | — | — | — |
| JP | 25 | F | 1 | — | — | — | — |
| HB | 46 | M | 1 | — | — | — | C & I |
| SP | 68 | M | 1 | — | — | — | C & I |
| JP | 35 | M | 1 | — | — | — | — |
| CR | 29 | F | 2 | 7 | — | — | — |
| JS | 36 | F | 2 | 30 | — | — | C & I |
| PS | 46 | F | 1 | — | — | — | B |
| KS | 62 | M | 1 | — | — | — | — |
| DT | 24 | M | 2 | — | — | — | C & I |
| VB | 71 | M | 2 | — | — | — | — |
| CA | 52 | M | 2 | — | — | — | B |
| AB | 50 | M | 1 | — | — | — | — |
| PC | 50 | M | 2 | — | — | — | — |
| CC | 55 | M | 2 | — | 5 | — | C & I |
| LH | 61 | F | 1 | — | — | — | — |
| AH | 65 | F | 2 | 48 | 5 | — | C & I |
| NK | 65 | F | 1 | — | — | — | — |
| CM | 29 | F | 1 | — | — | — | — |
| BS | 60 | M | 1 | — | — | — | — |
| AB | 40 | F | 1 | — | — | — | — |
| WF | 37 | M | 1 | — | — | — | — |
| EW | 62 | M | 1 | — | — | — | — |

C & I = chemoimmunotherapy with imidazole carboxamide and BCG vaccination.
B = immunotherapy with BCG vaccination.